# Hybrid Chaos via Entropy-Greedy Map Switching: A Statistical and Predictability Analysis

**DOI:** 10.3390/e28101071

**Published:** 2026-09-29

**Authors:** Alexandru Dinu, Corina Nitu

**Affiliations:** Faculty of Electronics, Telecommunications and Information Technology, National University of Science and Technology POLITEHNICA Bucharest, 060042 Bucharest, Romania; corina.macovei@upb.ro

**Keywords:** hybrid chaotic generator, entropy-greedy selection, pseudorandom number generation, dynamical analysis, security analysis, Lyapunov exponent, Kolmogorov–Smirnov test

## Abstract

We propose and analyse a hybrid chaotic pseudorandom generator that combines the logistic, tent, and Hénon maps under a deterministic entropy-greedy selector. At each step every map proposes a candidate; the selector keeps the one that maximises the plug-in Shannon entropy of a sliding output window, emits it, and advances only the winning map’s state. We benchmark all four generators plus a Mersenne Twister (MT19937) reference on uniformity (Kolmogorov–Smirnov statistic, KS), lag-correlation, entropy, Lyapunov exponent, and sensitivity. The hybrid reduces the logistic KS deviation by 26.9% and the maximum short-lag Pearson correlation by 6.5%. An explicit predictability analysis shows that the logistic and tent states are exposed, giving an adversary 92% one-step accuracy; only the hidden Hénon coordinate blocks free-running cloning, which diverges within about eight steps. Rank-uniformisation drops KS to $2.5\times 10^{-5}$ and passes a NIST SP 800-22 subset, while the lag-1 rank dependence is unchanged. Structural modifications—state masking for all component maps and continuous-time replacements with hidden coordinates—are discussed as routes to reducing predictability.

## 1. Introduction

Chaos-based generation of pseudorandom sequences has been studied for more than three decades [1,2,3]. The motivation is direct: chaotic maps exhibit sensitive dependence on initial conditions, dense periodic orbits, and topological mixing, properties that align with the confusion and diffusion requirements of symmetric cryptography [4,5].

Recent work has extended these ideas to high-dimensional memristive neural networks [6,7], to frequency-domain encryption via hyper-chaotic maps [8,9,10], and to composite pseudorandom generators built from coupled maps [11,12,13,14]. Deterministic finite-state automata driven by chaos have also been used to switch among simple 1D maps, yielding longer cycle lengths and broader chaotic ranges [15]. These developments underscore both the continuing interest in chaos-based cryptography and the need for quantitative characterisations of the generators that underpin it.

The logistic map, the tent map, and the Hénon map are among the most analysed systems in this context, with well-understood bifurcation diagrams, invariant measures, and Lyapunov spectra [16,17,18].

Single-map generators have known weaknesses. The logistic map at r=4 has an arcsine invariant density that concentrates probability mass near 0 and 1, so its raw output is non-uniform and detectable by simple statistical tests [19,20]. The tent map at μ=2 has a uniform marginal but its piecewise linearity makes symbolic dynamics easy to estimate [21]. The Hénon map adds a second dimension and a fractal attractor, but its scalar output must be folded back into [0,1], which distorts the marginal. Most importantly, a single deterministic map $x_{n+1}=f\left(x_{n}\right)$ can in principle be reconstructed from a sufficiently long output segment by exploiting the algebraic form of its recurrence [21,22,23].

Composite generators address part of this. Cascading two maps increases the effective state space and makes direct inversion more complex [24]. Hybrid schemes that alternate maps on a fixed schedule break the single-curve structure visible in a phase portrait [14]. When the schedule is fixed and public, however, an attacker can split the output into the sub-sequences produced by each map and invert them separately, so the gain is structural rather than fundamental.

In this paper the switching schedule is a function of the generator’s own output history rather than a fixed pattern. At each step all three maps propose a candidate; the selector keeps the candidate that maximises the plug-in Shannon entropy of the recent output window, advances only that map’s state, and emits the chosen value. The selector adds no free parameters beyond the window size *W* and needs no training.

Our aim is a careful and fair characterisation rather than a security claim, and the contributions are threefold:

1. A systematic comparison of the logistic, tent, Hénon, and hybrid generators on a single set of metrics. As a non-chaotic upper baseline, we include the Mersenne Twister (MT19937) [25], the default pseudorandom number generator (PRNG) of NumPy, Python, and the C++ standard library; it has period $2^{19937}-1$ and 623-dimensional equidistribution, is statistically excellent but not cryptographically secure, and therefore marks the quality level a chaotic generator should be measured against.
2. An explicit predictability analysis. We show that the logistic and tent internal states are exposed in the output, that an adversary with the public model predicts the next sample with probability $1-p_{H\overset{´}{e}\mathrm{non}}$ under continuous observation, and that only the hidden Hénon coordinate blocks exact free-running cloning. We discuss structural modifications—state masking applied to all three component maps and continuous-time replacements with genuinely hidden coordinates—that could lower the prediction rate.
3. A post-processing study. The raw hybrid output is non-uniform (Kolmogorov–Smirnov statistic, hereafter KS, of 0.087), so it cannot serve directly as a key stream. We apply the empirical rank transform, which forces the marginal to be exactly uniform by construction, reducing KS to $2.5\times 10^{-5}$. The uniformised bit stream passes a three-test subset of the National Institute of Standards and Technology (NIST) Special Publication 800-22 suite [26] (monobit, block frequency, and runs) at significance α=0.01. However, rank-uniformisation is a monotone transform, so it leaves the lag-1 Spearman rank correlation unchanged at −0.146. We discuss low-latency whitening alternatives suitable for real-time generation and note that a full NIST battery and the TestU01 suite [27] should follow a dedicated decorrelation stage, as recommended in [8,9].

The remainder of the paper is organised as follows. Section 2 introduces the maps, the entropy estimator, and the Lyapunov criterion. Section 3 defines the generator, its computational cost, and the predictability analysis. Section 4 reports all numerical experiments. Section 5 discusses scope and limitations, and Section 6 concludes.

## 2. Background

This section defines the three component maps, the statistical metrics used throughout, and the Lyapunov exponent estimation procedure.

### 2.1. Discrete Chaotic Maps

Let $x_{n}\in \left(0,1\right)$ throughout. Each of the three maps below has a well-characterised bifurcation diagram in the literature [16,17,18]; we operate each map at a single parameter set deep inside its chaotic regime, so a full bifurcation scan is not repeated here. We do, however, verify the chaotic nature via a positive Lyapunov exponent estimated from 50 uniformly sampled initial conditions (Section 2.3).

Logistic map.

(1) $$x_{n+1}=r\;x_{n}\left(1-x_{n}\right),\;r\in \left(0,4\right].$$

At r=4 the system is fully chaotic with Lyapunov exponent ln2 per iteration (i.e., 1 bit) and invariant density $\rho \left(x\right)=1/\left(\pi \sqrt{x(1-x)}\right)$ [16]. This arcsine density is strongly non-uniform. We use r=3.99 to stay in the chaotic regime while avoiding the degenerate band-merging endpoint. The logistic map has been used as a key-stream source in many encryption schemes [1,28], with its statistical shortcomings documented by Li et al. [19].

Tent map.

(2) $$x_{n+1}=\left\{\begin{array}{cc} \mu \;x_{n} & x_{n}<\frac{1}{2},\\ \mu (1-x_{n}) & x_{n}\geq \frac{1}{2}, \end{array}\right.\;\mu \in \left(0,2\right].$$

At μ=2 the invariant measure is Lebesgue on [0,1], giving a uniform marginal distribution. Concretely, the uniform density is a fixed point of the transfer (Perron–Frobenius) operator: each output value has two preimages and $|f^{\prime }|=2$ everywhere, so a flat input density maps to a flat output density. On a bounded interval the uniform law is the maximum-entropy distribution [29], so this is the best a single one-dimensional map can achieve. Its piecewise-linear form, however, makes symbolic-dynamics estimation straightforward [21]. We use μ=1.99.

Hénon map.

(3) $$\left(\begin{array}{c} x_{n+1}\\ y_{n+1} \end{array}\right)=\left(\begin{array}{c} 1-a\;x_{n}^{2}+y_{n}\\ b\;x_{n} \end{array}\right),\;a=1.4,\;\;b=0.3.$$

The Hénon attractor has Hausdorff dimension ≈1.26 and a positive Lyapunov exponent ≈0.42 nats per iteration [17,30]. We use the *x*-component folded as |x|mod1 for the scalar output. Because the attractor concentrates on thin filaments and does not fill the unit square, the folded output inherits this clustering and its histogram is non-uniform (KS distance from the uniform law 0.113, Table 2). The second coordinate *y* is never emitted; this fact is central to the predictability analysis in Section 3.3.

### 2.2. Statistical Metrics: Independence, Uniformity, and Entropy

Independence of successive outputs is necessary for cryptographic suitability. We quantify it with the sample Pearson correlation at lag *k*,

(4) $$\rho_{k}=\frac{\sum_{n=1}^{N-k}\left(x_{n}-\bar{x}\right)\left(x_{n+k}-\bar{x}\right)}{\sum_{n=1}^{N}{\left(x_{n}-\bar{x}\right)}^{2}},$$

which should be near zero for all k≥1. The sample autocorrelation function (ACF) at lag *k* is $r_{k}=\rho_{k}$ of Equation (4); Figure 5 plots the signed $r_{k}$ over many lags and Figure 6 plots $|\rho_{k}|$ over short lags. Under the white-noise null hypothesis, the values lie inside the band $\pm 1.96/\sqrt{N}$ [31].

Uniformity of the marginal is measured by the one-sample Kolmogorov–Smirnov (KS) statistic against the uniform law on [0,1],

(5) $$D_{N}=\underset{x\in [0,1]}{\sup}\left|{\hat{F}}_{N}\left(x\right)-x\right|,$$

where ${\hat{F}}_{N}$ is the empirical cumulative distribution function of the output; smaller $D_{N}$ means a more uniform output.

The selector uses a sliding-window plug-in Shannon estimate. For the window $\left\{x_{n-W+1},\ldots ,x_{n}\right\}$ binned into B=16 equal-width intervals over [0,1],

(6) $${\hat{H}}_{n}=-\sum_{k=1}^{B}{\hat{p}}_{k}\log_{2}{\hat{p}}_{k},\;{\hat{p}}_{k}=\frac{c_{k}+\varepsilon }{\sum_{j}\left(c_{j}+\varepsilon \right)},$$

with $c_{k}$ the bin count and $\varepsilon =10^{-12}$ a smoothing constant. The maximum is $\log_{2}B=4$ bits. The plug-in estimator is negatively biased for small samples: with W=50 and B=16 there are on average about three counts per bin, so lightly populated bins systematically underestimate the true entropy. The Miller–Madow correction $\hat{H}+\left(\hat{B}-1\right)/\left(2W\ln2\right)$, where $\hat{B}$ is the number of occupied bins, removes the leading bias term [29,32]. We verified that replacing the plug-in estimator with the Miller–Madow corrected version inside the selector loop does not change the winning map on any of the 20,000 steps in our default run: the bias is common to all three candidates and cancels in the argmax, so the selection dynamics are unaffected. On a single W=50 hybrid window we measure a plug-in value of 3.78 bits against a Miller–Madow value of 4.00 bits. We therefore use the plug-in estimate only as a relative selection criterion and report mean entropies (Section 4) at the larger diagnostic window W=100, where the per-bin count—and hence the bias—is smaller.

### 2.3. Lyapunov Exponent

The largest Lyapunov exponent λ measures the mean exponential rate of divergence of nearby trajectories, $\lambda =\int \log_{2}\left|f^{\prime }\left(x\right)\right|\;d\mu \left(x\right)$ for a scalar map *f* with invariant measure μ [33]. For the hybrid, where the active map changes each step, *f* is not fixed, so we estimate λ numerically: we run two trajectories started a distance $\varepsilon =10^{-9}$ apart, average over 50 uniformly sampled initial conditions, and fit the slope of $\log_{2}\left|x_{n}-x_{n}^{\prime }\right|$ in the initial linear-growth region before the error saturates at O(1) [18]. The standard deviation across the 50 runs is reported alongside the mean in Figure 9. A formal analytical bound on the Lyapunov exponent for a switched-map system remains an open problem; the numerical estimate, while not a proof, is consistent with the exponential divergence observed in Figure 8 and with the known positivity of λ for each component map at the chosen parameters [16,17].

## 3. Hybrid Generator

This section defines the generator architecture (Section 3.1), analyses its computational cost (Section 3.2), and presents the predictability analysis that scopes its security limitations (Section 3.3).

### 3.1. Architecture and State Update

The generator keeps three internal states: $s^{\left(L\right)}$ for the logistic map, $s^{\left(T\right)}$ for the tent map, and $\left(s_{x}^{\left(H\right)},s_{y}^{\left(H\right)}\right)$ for the Hénon map. At step *n* each map proposes a candidate,

(7) $$\begin{array}{cc} c^{\left(L\right)} & =f_{L}\left(s^{\left(L\right)}\right)\;\mathrm{mod}\;1, \end{array}$$

(8) $$\begin{array}{cc} c^{\left(T\right)} & =f_{T}\left(s^{\left(T\right)}\right)\;\mathrm{mod}\;1, \end{array}$$

(9) $$\begin{array}{cc} c^{\left(H\right)} & =\left|f_{H,x}\left(s_{x}^{\left(H\right)},s_{y}^{\left(H\right)}\right)\right|\;\mathrm{mod}\;1. \end{array}$$

For the logistic and tent maps with our parameters, the raw image stays below 1, so the fold in (7) and (8) is inactive; the fold matters only for the Hénon candidate. The selector evaluates the “what-if” window entropy for each candidate, defined as

(10) $$\hat{H}\left(c\right)\;=\;\hat{H}\left(\{x_{n-W+1},\;\ldots ,\;x_{n-1},\;c\}\right),$$

i.e., the plug-in Shannon entropy (Equation (6)) computed on the window obtained by tentatively appending the candidate *c* to the W−1 most recent outputs. The selector then chooses

(11) $$k^{*}=\arg\max_{k\in \{L,T,H\}}\hat{H}\;\left(c^{\left(k\right)}\right).$$

The output is $x_{n}=c^{\left(k^{*}\right)}$, and only the winning map advances. The update rule, left implicit in similar work, is made explicit here:

(12) $$s^{\left(L\right)}\leftarrow c^{\left(L\right)},\;s^{\left(T\right)}\leftarrow c^{\left(T\right)},\;\left(s_{x}^{\left(H\right)},s_{y}^{\left(H\right)}\right)\leftarrow \left(1-a\;{\left(s_{x}^{\left(H\right)}\right)}^{2}+s_{y}^{\left(H\right)},\;b\;s_{x}^{\left(H\right)}\right),$$

where only the line for $k^{*}$ is applied. Two consequences follow and are used later. First, for the logistic and tent maps the new state equals the emitted value, so each of those states is exposed in the output stream. Second, a losing map keeps its state, so its candidate is unchanged until it wins; at any step at most one of the three candidates is new. Introducing a small perturbation to losing maps’ states (e.g., advancing them by a fraction of an iteration) could increase long-term complexity; this remains to be evaluated. Algorithm 1 summarises the procedure.
**Algorithm 1** Hybrid entropy-greedy chaotic generator**Require:** 
Initial states $x_{0}^{\left(L\right)},\;x_{0}^{\left(T\right)},\;\left(x_{x,0}^{\left(H\right)},x_{y,0}^{\left(H\right)}\right)$; window size *W*; length *N*.**Ensure:** 
Sequence ${\left\{x_{n}\right\}}_{n=1}^{N}$.1:$\mathrm{history}\leftarrow [x_{0}^{\left(L\right)}]$2:**for** n=1 **to** *N* **do**3:    Compute $c^{\left(L\right)},c^{\left(T\right)},c^{\left(H\right)}$ via Equations (7)–(9)4:    $k^{*}\leftarrow \arg\max_{k}\;\hat{H}\left(c^{\left(k\right)}\right)$                // Equation (10)5:    $x_{n}\leftarrow c^{\left(k^{*}\right)}$; advance the state of map $k^{*}$ via Equation (12)6:    Append $x_{n}$ to history7:**end for**

The criterion is a one-step greedy policy over an ensemble of three maps. We do not claim a connection to bandit or reservoir methods beyond this: there is no reward signal, no readout, and no training—the selector is an analytic, parameter-free rule.

### 3.2. Computational Cost and Practical Timing

The secret is the initial condition $\left(x_{0}^{\left(L\right)},x_{0}^{\left(T\right)},x_{x,0}^{\left(H\right)},x_{y,0}^{\left(H\right)}\right)$; the map parameters and the selection rule are assumed public, as is standard. Each iteration requires three map evaluations and three entropy estimates over a window of length *W*. Histogramming is O(W), the entropy sum is O(B), and the argmax is O(1), so the per-step cost is O(W) with B=16 constant.

Table 1 reports wall-clock times for the main experiments on a single core of an Intel Core i7-12700H at 2.3 GHz running Python 3.11/NumPy 1.26. The N=20,000 default run (all five generators plus diagnostics) completes in under 8 s; the Lyapunov sweep over ten values of *W* with 50 initial conditions each takes ∼42 s. For W=50 and $N=10^{6}$, the hybrid alone runs in about 45 s, acceptable for off-line generation.

### 3.3. Predictability Analysis

We now turn to the central security question. A fixed-map generator $x_{n+1}=f\left(x_{n}\right)$ has a public recurrence and a single secret $x_{0}$; from a block of output an adversary sets up an inversion problem with one unknown. It is tempting to argue that, because the switching sequence of the hybrid depends on the output, reconstructing it “is equivalent to predicting the output” and so confers unpredictability. This argument is incorrect, and we make the actual situation precise.

#### 3.3.1. State Exposure

By Equation (12), whenever the logistic map wins, the emitted value equals its new state; likewise for the tent map. Hence two consecutive logistic wins at positions a<b (with no logistic win in between) must satisfy $x_{b}=f_{L}\left(x_{a}\right)$ exactly, and similarly for the tent map. We verify this on N=20,000 samples: across all 15,204 consecutive logistic-win pairs and 3173 consecutive tent-win pairs, the maximum deviation is 0 to machine precision. Labelling each output by its source is trivial once the two static (frozen) candidates are known: the adversary checks whether the emitted sample matches the stored $c^{\left(L\right)}$ or $c^{\left(T\right)}$; if it matches neither, Hénon won. The adversary therefore recovers $s^{\left(L\right)}$ and $s^{\left(T\right)}$ exactly and can compute $c^{\left(L\right)}$ and $c^{\left(T\right)}$ at every future step. The only state not exposed is the Hénon *y*-coordinate, which is never emitted.

#### 3.3.2. Threat Model 1: One-Step Prediction Under Observation

This is the relevant model when the output is used as a key stream and the adversary observes it. The adversary computes the two known candidates and predicts the one with higher what-if window entropy (Equation (10)), using the true observed window. Because the generator’s argmax over {L,T,H} equals its argmax over {L,T} on every step where Hénon is not the winner, and the adversary evaluates that same restricted argmax on the same window, its prediction is exact whenever the logistic or tent map wins and wrong only when the Hénon map wins. The one-step accuracy is thus $1-p_{H\overset{´}{e}\mathrm{non}}$. Empirically, over 8000 steps we measure 0.9175 with $p_{H\overset{´}{e}\mathrm{non}}=0.0825$; even a trivial “always emit $c^{\left(L\right)}$” predictor reaches 0.738 (Figure 1b).

#### 3.3.3. Threat Model 2: Free-Running Clone

Here the adversary fixes the recovered $s^{\left(L\right)},s^{\left(T\right)},s_{x}^{\left(H\right)}$ at a split point but must guess the hidden *y*, then rolls the generator forward without further observation. With a wrong *y* the clone tracks the true sequence only until the Hénon candidate first changes the argmax; the sensitive dependence then amplifies the mismatch and the trajectories separate. We observe divergence within about 8 steps (Figure 1a).

#### 3.3.4. Structural Mitigations

Two modifications could reduce the 92% one-step prediction rate. First, hashing every candidate—not only the Hénon one—through a lightweight mixing function before emission (e.g., XOR-folding with a secret nonce) would hide the identity of the winning map and break the direct correspondence between emitted value and internal state for all three component maps, making the labelling step of the attack infeasible without the nonce. Second, replacing the logistic and tent maps with continuous-time systems whose observable output is a strict subset of their state—such as the *x*-component of the Lorenz or Rössler system [5]—would ensure that no emitted sample fully reveals the component state. We leave quantitative evaluation of both modifications to future work, but note that they address the root cause identified by the analysis above: full state exposure of the logistic and tent maps.

The hybrid raises the bar over a single invertible map and resists exact state cloning through one hidden coordinate, but it does not deliver keystream unpredictability. Its merit is statistical and structural, quantified next. Any cryptographic use must add a uniformisation and decorrelation stage (Section 4.8); the raw output is not a key stream.

In Figure 1a the vertical axis is the absolute error $|x_{n}-{\hat{x}}_{n}|$ on a log scale; it stays at $∼10^{-16}$ until a Hénon win injects the wrong *y* around step 8, after which the positive Lyapunov exponent drives it to O(1). In Figure 1b the running prediction accuracy converges to ≈0.92; the dotted line marks the analytic ceiling $1-p_{H\overset{´}{e}\mathrm{non}}$.

## 4. Numerical Results

All experiments use N=20,000 iterations (N=5000 for the scatter/ACF figures) with initial conditions $x_{0}=0.4732$, $y_{0}=0.3141$, selector window W=50, and diagnostic entropy window W=100 unless stated otherwise. The logistic, tent, and Hénon maps share $x_{0}$. The Mersenne Twister reference is generated with NumPy’s MT19937 engine on the same sample size. All reported statistics are deterministic given the initial conditions; where variance across runs matters (Lyapunov estimation), we report standard deviations over 50 independent initialisations.

### 4.1. Phase Portraits

The delay-1 portrait $\left(x_{n},x_{n+1}\right)$ reveals a single map’s functional form as a one-dimensional curve.

A clean curve in Figure 2 means the next value is a deterministic function of the current one, hence trivially predictable. The logistic map traces the parabola $x^{\prime }=rx\left(1-x\right)$, the tent map the Λ-shaped curve, and the Hénon map a thickened fractal curve. The hybrid (panel d) scatters many points off these curves, so it no longer concentrates on a single one-dimensional set. It does not, however, become a featureless cloud: the logistic parabola and the tent ridges remain partly visible, because two consecutive logistic (or tent) wins still place the pair $\left(x_{n},x_{n+1}\right)$ exactly on that map’s curve. This residual structure is the visual counterpart of the high one-step predictability of Section 3.3. Around x≈0.4–0.5 the scatter is densest because the logistic parabola peaks there and the tent map folds, so both maps’ candidates cluster in that region.

### 4.2. Sliding-Window Entropy

Figure 3 traces ${\hat{H}}_{n}$ (Equation (6)) over time; a higher, flatter curve closer to the 4-bit ceiling indicates a more uniform recent window. The tent map has the highest mean entropy (3.879 bits), followed by the Hénon map (3.844), the hybrid (3.808), and the logistic map (3.745); the Mersenne Twister gives 3.885. The hybrid thus exceeds only the logistic baseline on this measure. The selector improves on the logistic map by pulling the running window towards uniformity, but the Hénon map’s folded marginal is already closer to uniform on average, so its mean window entropy is higher than the hybrid’s even though Hénon is selected only 8% of the time (Section 4.3).

### 4.3. Map Selection Statistics

Figure 4a shows which map is active at each step; Figure 4b gives the aggregate counts. The logistic map is selected 76.0% of the time, the tent map 15.9%, and the Hénon map 8.1%. The asymmetry follows from the marginals, not the mean entropy: the logistic arcsine density places mass towards the edges 0 and 1, which are exactly the bins the other two maps tend to leave under-filled, so the logistic candidate wins the one-step entropy contest most often—despite having the lowest long-run mean. A penalty or rebalancing term is a natural extension (Section 5). No map dominates for more than a few consecutive steps.

### 4.4. Autocorrelation and Lag-Correlation

Figure 5 plots the signed sample ACF; values inside the dashed band are statistically indistinguishable from white noise. All chaotic generators have ACF values near zero, consistent with mixing. The Hénon map shows the largest excursions beyond the band. The hybrid shows no systematic structure and is broadly similar to the tent map.

Figure 6 plots $|\rho_{k}|$ over lags 1–20; lower is better. The tent map has the lowest correlations at all lags. At lag 1 the logistic and Hénon maps give $|\rho_{1}|=0.134$ and 0.168; the hybrid gives 0.125, a 6.5% reduction over the logistic baseline. None of the four chaotic generators reaches $|\rho_{1}|<1.96/\sqrt{N}$—a known limitation of deterministic maps without post-processing [22,31]—whereas the Mersenne Twister gives $|\rho_{1}|=0.007$, inside the band. A dedicated decorrelation (whitening) stage is therefore necessary before any cryptographic use; see Section 4.8.

### 4.5. Marginal Distributions

Figure 7 shows the marginal of each generator; the closer to the flat dashed line, the smaller the KS statistic (Equation (5)). The logistic map shows the U-shaped density, the tent map is closest to uniform, and the Hénon map has a non-trivial folded distribution. The hybrid suppresses the U-shape relative to the logistic baseline but does not reach the tent map’s uniformity, since the logistic map supplies 76% of the output. Table 2 collects the figures of merit.

The hybrid’s advantage over the individual chaotic maps is structural (mixed functional form, one hidden coordinate) rather than statistical dominance: the tent map alone is better on every column except structural invertibility. The Mersenne Twister is better than every chaotic generator on every column and quantifies the gap that post-processing must close.

### 4.6. Sensitivity to Initial Conditions

Figure 8 plots $|x_{n}-x_{n}^{\prime }|$ on a log scale; a straight rising segment indicates exponential divergence (positive Lyapunov exponent) and the plateau is saturation at O(1). Both the logistic map and the hybrid diverge exponentially and saturate with a similar onset, confirming that switching does not suppress chaos.

### 4.7. Lyapunov Exponent Versus Window Size

Figure 9(left) reports the numerical Lyapunov exponent of the hybrid as *W* varies; shaded bands show ±1 standard deviation over 50 initial conditions. The logistic reference ≈1.03 is dashed. The hybrid exponent is positive for every W∈[10,200], in the range 0.32–0.52 bits per iteration. As *W* grows the logistic share rises towards 0.97 (right panel) and the exponent decreases because the hybrid behaves increasingly like a sub-sampled logistic map.

The choice W=50 balances three considerations: (i) map diversity—at W=50 the logistic share is 76%, tent 16%, Hénon 8%, whereas at W≥100 the logistic share exceeds 93% and the structural benefit of switching diminishes; (ii) entropy estimation quality—at W=10 each bin has fewer than one count on average, making the plug-in estimate noisy, while at W=50 there are ∼3 counts per bin; (iii) computational cost—per-step cost is O(W). We therefore adopt W=50 as the default.

### 4.8. Post-Processing: Uniformisation and a NIST Subset

The KS column of Table 2 shows the raw hybrid output is not uniform, so it cannot serve as a key stream directly. We apply the empirical rank transform $u_{i}=\left(\mathrm{rank}\left(x_{i}\right)-\frac{1}{2}\right)/N$, which yields an exactly uniform marginal by construction.

Figure 10 contrasts the raw histogram (a) with the rank-transformed one (b), which is flat by construction. The KS statistic falls from 0.087 to $2.5\times 10^{-5}$. The Pearson lag-1 correlation changes from $\rho_{1}=-0.125$ (raw) to $\rho_{1}=-0.122$ (uniformised)—a negligible shift, since the rank transform is monotone and nearly preserves linear correlation. The Spearman rank correlation is exactly preserved at $r_{s}=-0.146$ before and after, confirming that the serial dependence structure is untouched.

To produce a binary stream for the NIST tests, each uniformised sample $u_{i}\in \left[0,1\right)$ is mapped to a 16-bit unsigned integer via $b_{i}=\left\lfloor u_{i}\cdot 2^{16}\right\rfloor$ and its bits are concatenated in big-endian order, yielding N×16=320,000 bits for N=20,000. We run a subset of NIST SP 800-22 [26] at significance α=0.01 (Table 3).

Each entry is the test’s *p*-value; the stream fails when p<α. The raw hybrid’s $p=1.7\times 10^{-21}$ on monobit and p=0 on runs are decisive failures. After uniformisation all three tests pass. The block-frequency p=1.000 passes trivially because the rank transform enforces a balanced marginal; only the order-sensitive runs test carries non-trivial information. For a comprehensive evaluation, recent works [8,9] recommend the full 15-test NIST battery and the TestU01 suite [27], which should be applied after a dedicated whitening stage addresses the residual lag dependence; we defer both to future work.

The rank transform requires sorting the entire sequence before emission, introducing O(NlogN) latency. Two lower-latency alternatives merit investigation: (i) a sliding empirical CDF that maps each sample through a running histogram in O(B) per step; (ii) an XOR whitener, $z_{n}=q_{n}⊕q_{n-d}$ for a fixed delay *d*, to break short-lag correlation at negligible cost.

## 5. Discussion

### 5.1. Comparison with Fixed Composite Maps

A fixed alternating schedule breaks the single-curve portrait but leaves the schedule public, so the output splits into per-map sub-sequences that are inverted independently. The output-dependent schedule here removes the *fixed* public pattern, but Section 3.3 shows it does not remove predictability: the schedule is itself a public function of the observed output, so an adversary reproduces it. The actual gain over a fixed composite is the absence of a static schedule to exploit, plus one hidden coordinate that blocks free-running cloning—not unpredictability.

### 5.2. Why Not the Tent Map Alone

Table 2 shows the tent map beats the hybrid on KS deviation and lag-correlation, so on statistics alone the tent map at μ→2 is the better single choice. The case for the hybrid is structural: the tent map’s piecewise-linear form makes its symbolic dynamics easy to estimate from output [21], whereas combining a quadratic, a piecewise-linear, and a two-dimensional quadratic map leaves no single functional form to invert. This structural argument holds in full only for the Hénon component, since the logistic and tent states are individually exposed. We claim the structural benefit only for the part of the output that the hidden Hénon coordinate protects.

### 5.3. Limitations

Three limitations are intrinsic. (i) The marginal is non-uniform (Table 2); raw output needs uniformisation, and uniformisation alone does not remove lag dependence (Section 4.8). (ii) The selection is strongly skewed toward the logistic map, more so as *W* grows (Figure 9); a rebalancing term—for instance subtracting a usage penalty $\beta \cdot n_{k}/n$ from the window entropy of map *k*, where $n_{k}$ is its cumulative selection count and β is a tunable parameter—would discourage over-use of any single map and could raise Hénon’s share above the current 8%, increasing both structural diversity and the effective hidden-state size. (iii) The security analysis is empirical and model-specific; a formal bound on an adversary’s success probability is open.

Under finite-precision (fixed-point) arithmetic, chaotic orbits inevitably become periodic. For the logistic map at r=3.99 in IEEE 754 double precision, the expected cycle length is of order $2^{52/2}\approx 6.7\times 10^{7}$ iterations [22]; the hybrid, which switches among three maps, could either extend or shorten this depending on the interaction of their respective cycle structures. A systematic finite-precision cycle-length study is necessary before any hardware deployment.

### 5.4. Comparison with Recent Composite Generators

Several recent composite chaos-based PRNGs are relevant. Murillo-Escobar et al. [11] proposed a 2D Hénon-Sine hyperchaotic map enhanced with a remainder function and demonstrated improved uniformity on a microcontroller. Patidar and Singh [12] used Hamiltonian conservative chaotic systems to build a PRNG with superior phase-space coverage. Zhao et al. [13] combined a chaotic map with quantum random walks for a hybrid PRNG. Erkan et al. [14] designed a 2D Rosenbrock map via optimisation benchmark functions. Alawida et al. [15] introduced deterministic chaotic finite-state automata to switch among 1D maps, achieving broader chaotic ranges and longer cycle lengths. Compared with these, our contribution is not a new chaotic map but a deterministic, parameter-free switching rule together with an explicit predictability analysis that quantifies what the construction achieves and what it does not. We regard this transparency as complementary to the design innovations cited above.

### 5.5. Relation to Prior Work

The improved lag-correlation of the hybrid would translate into a more uniform permutation in the pixel-shuffling scheme of [28]. The observability and independence analyses of Lorenz and Rössler systems in [5,31] suggest continuous-time systems with a genuinely hidden coordinate as better component candidates than the maps used here, precisely because the predictability analysis identifies hidden state as the only quantity that resisted reconstruction.

## 6. Conclusions

We have characterised a hybrid chaotic generator that selects, at each step, the map whose candidate maximises a sliding-window Shannon entropy, advancing only the winner. On a common benchmark against the logistic, tent, and Hénon maps and a Mersenne Twister reference, the hybrid is second among the chaotic generators on uniformity, reduces the logistic KS deviation by 26.9% and the maximum short-lag Pearson correlation by 6.5%, and keeps a positive Lyapunov exponent for all window sizes tested.

We deliberately scoped the security question rather than asserting it. The logistic and tent states are exposed in the output, so an adversary with the public model predicts the next sample with accuracy $1-p_{H\overset{´}{e}\mathrm{non}}\approx 0.92$ under observation; only the hidden Hénon coordinate blocks exact free-running cloning. The construction thus offers statistical and structural improvement over the logistic map and resistance to state cloning, but not keystream unpredictability. Rank-uniformisation removes the marginal defect (KS $\to 2.5\times 10^{-5}$) and passes a NIST subset, while the lag-1 rank dependence is unchanged, delimiting what post-processing alone achieves.

Several concrete directions follow. First, a decorrelation stage—XOR whitening or a sliding empirical CDF—should target the residual lag-1 dependence, after which the full NIST SP 800-22 and TestU01 [27] batteries become meaningful. Second, a rebalancing penalty in the selector would reduce the logistic skew and raise map diversity. Third, state masking applied to all component maps and continuous-time replacements with genuinely hidden coordinates [5] are routes to lowering the 92% prediction rate. Fourth, a formal bound on state-reconstruction complexity should replace the current empirical analysis. Fifth, a finite-precision cycle-length study under fixed-point arithmetic is a prerequisite before any hardware implementation, since finite precision can shorten the period sharply [22]. Sixth, perturbing losing maps’ states at each step could increase structural complexity and is worth evaluating alongside the rebalancing mechanism.

## Figures and Tables

**Figure 1:**
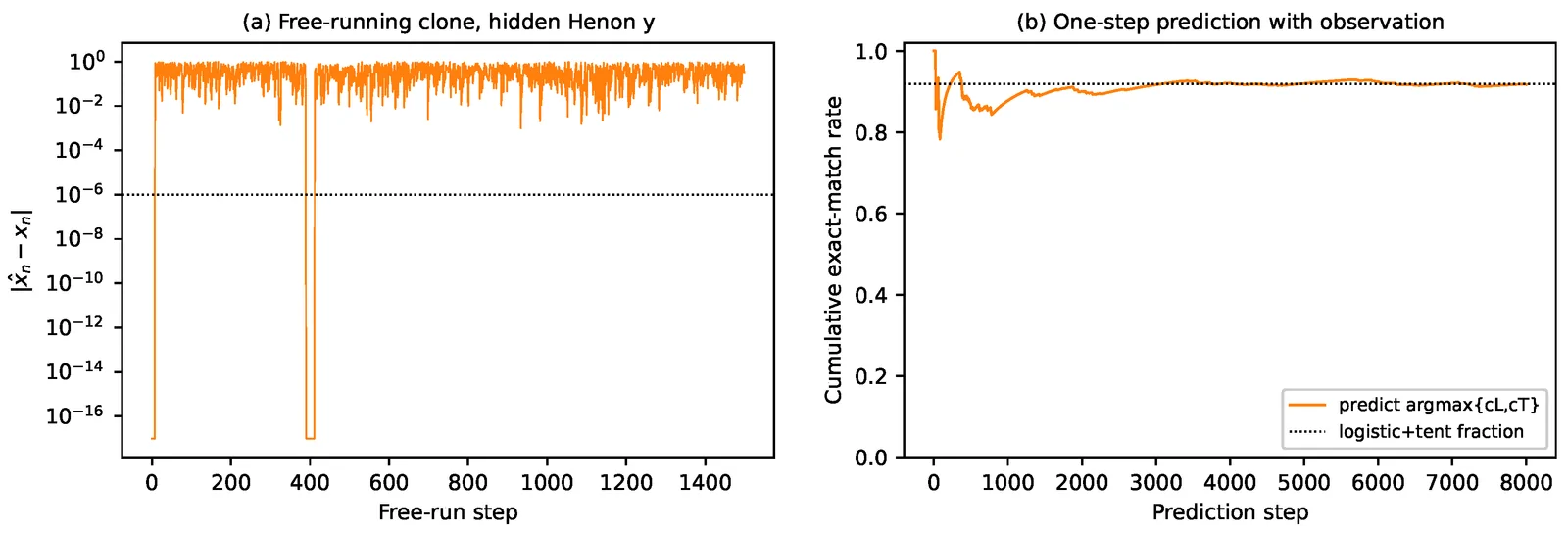
Reconstruction attacks.

**Figure 2:**
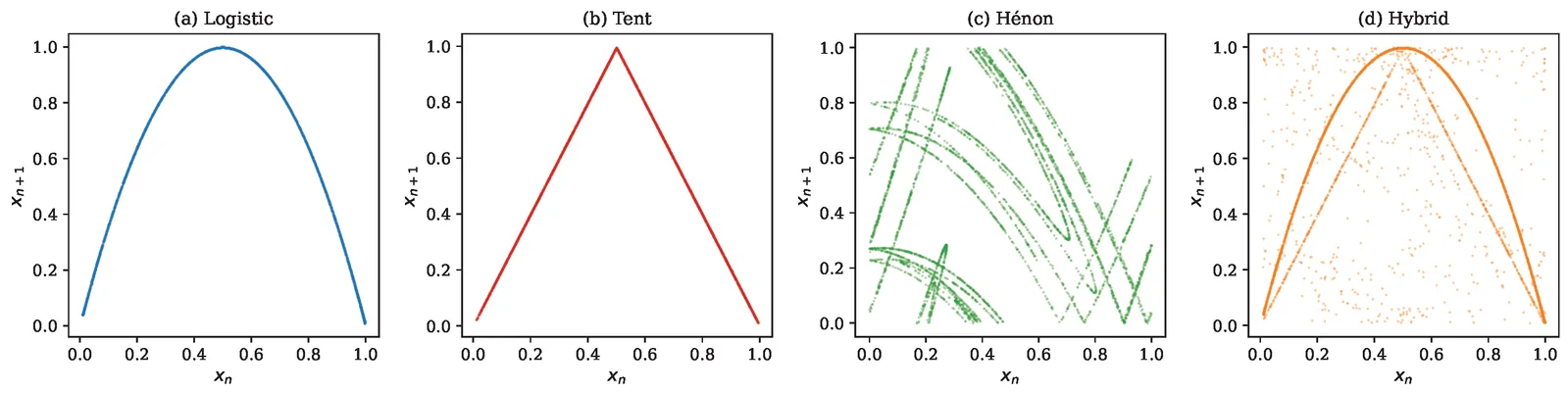
Delay-1 phase portraits, N=5000.

**Figure 3:**
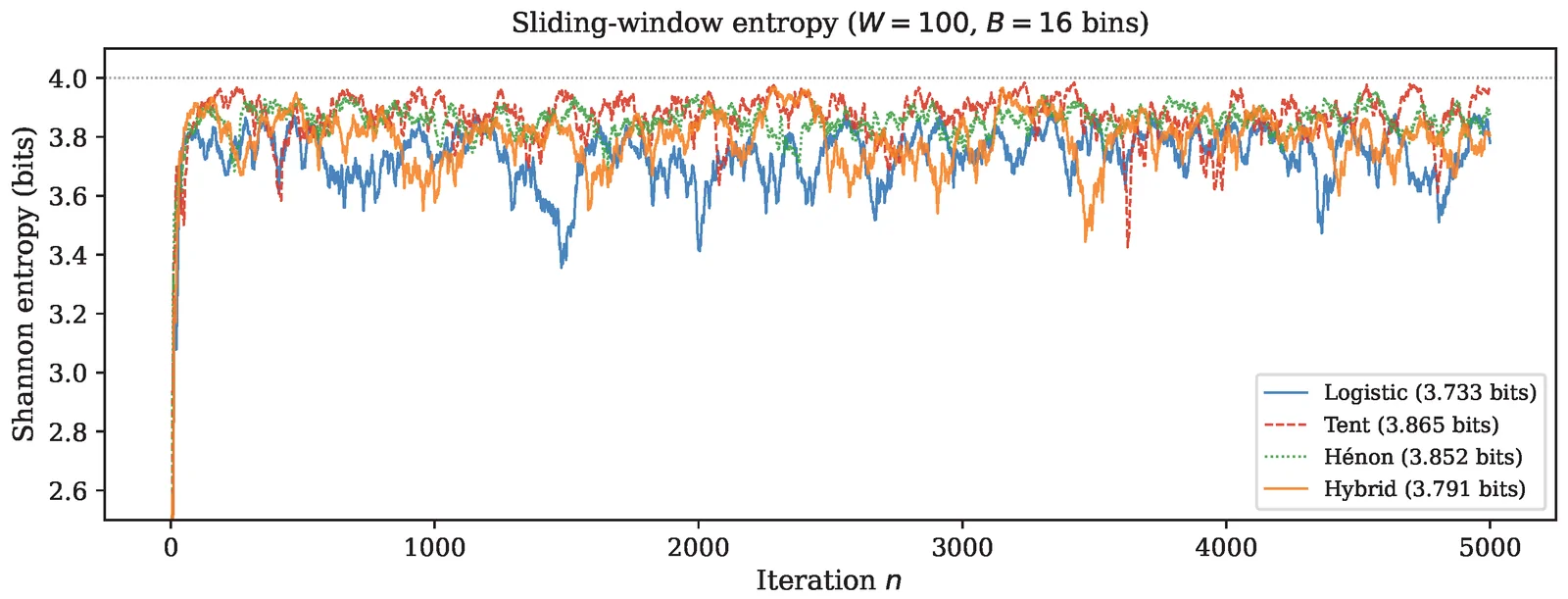
Sliding-window entropy (parameters W=100, B=16); dashed: means.

**Figure 4:**
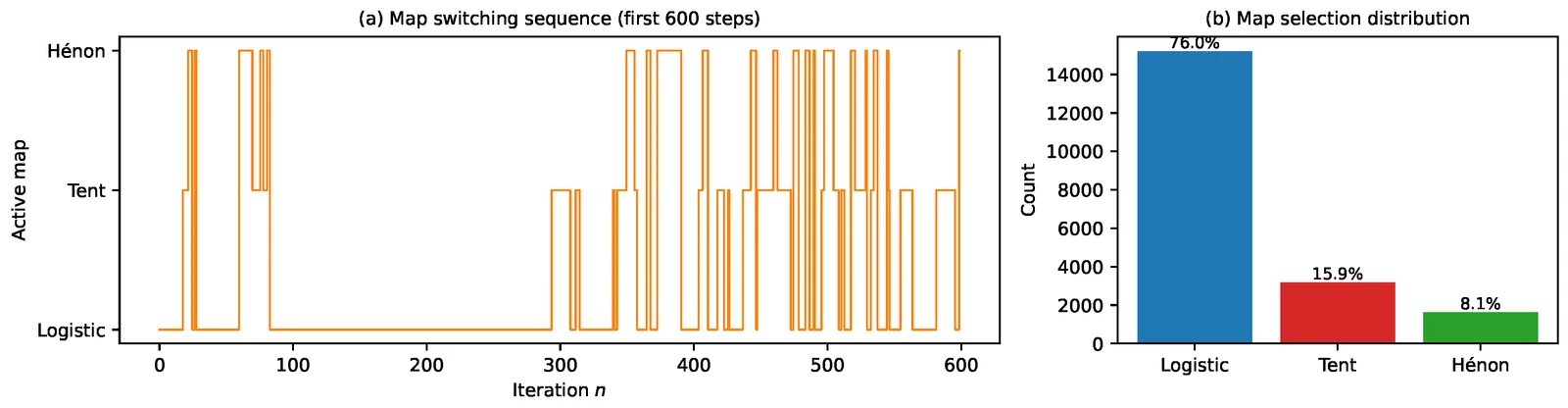
Map switching (first 600 steps) and selection counts, *N* = 20,000.

**Figure 5:**
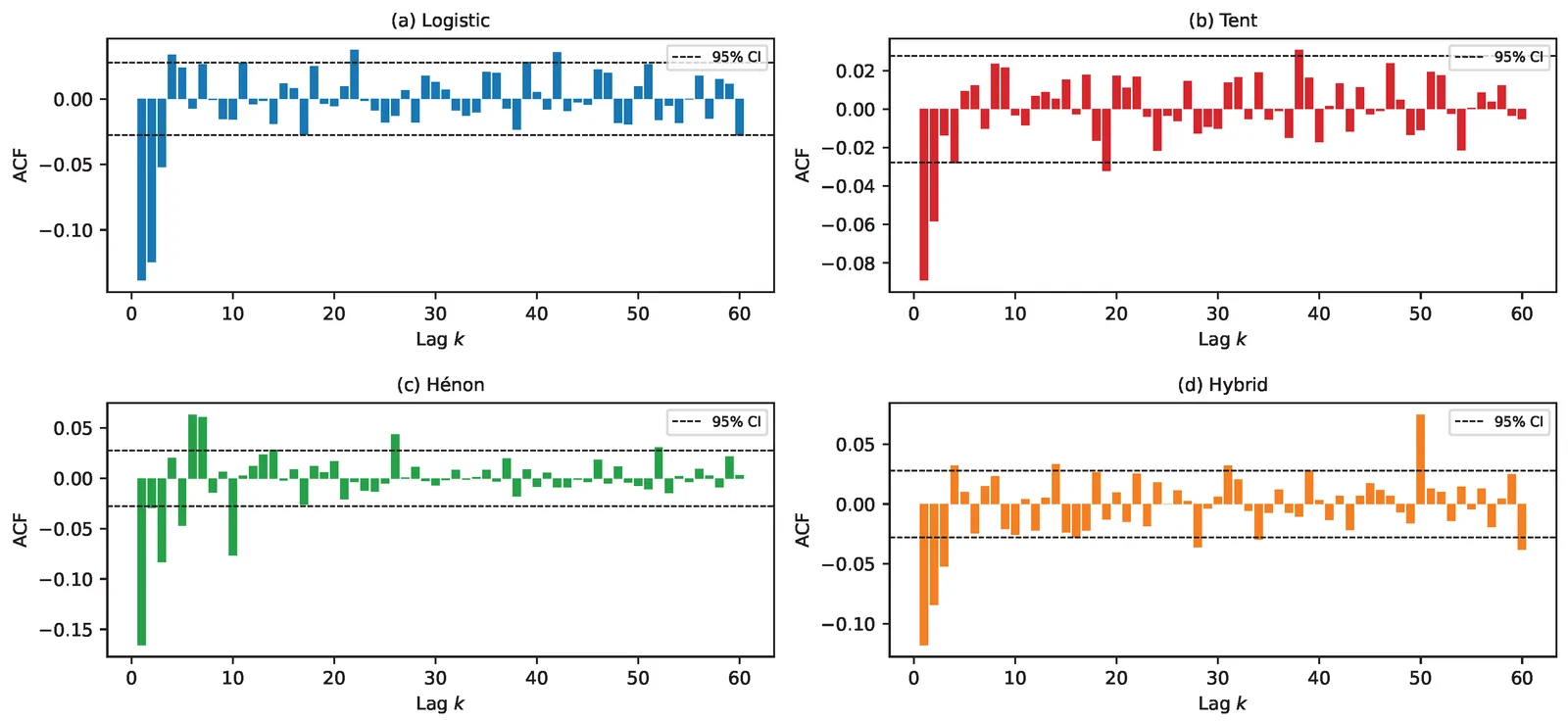
ACF, N=5000; dashed: 95% band.

**Figure 6:**
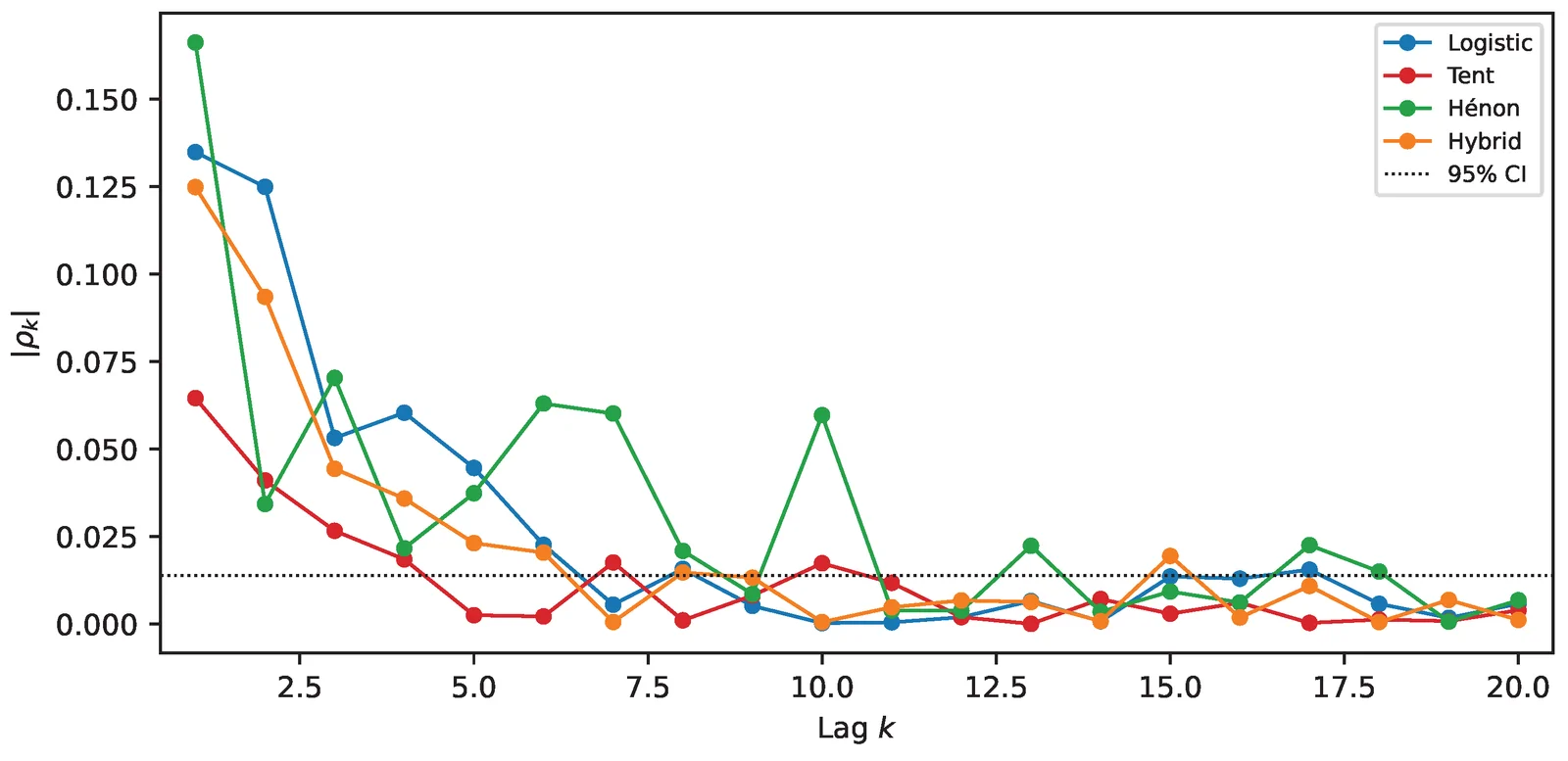
$|\rho_{k}|$, *N* = 20,000; dotted: 95% band.

**Figure 7:**
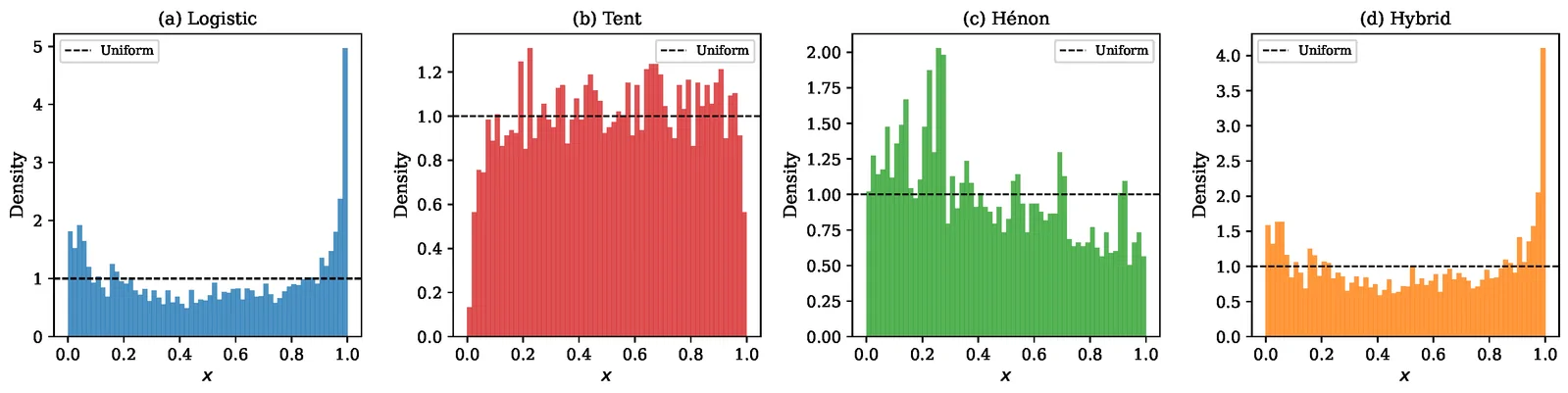
Histograms (60 bins, N=5000); dashed: uniform density.

**Figure 8:**
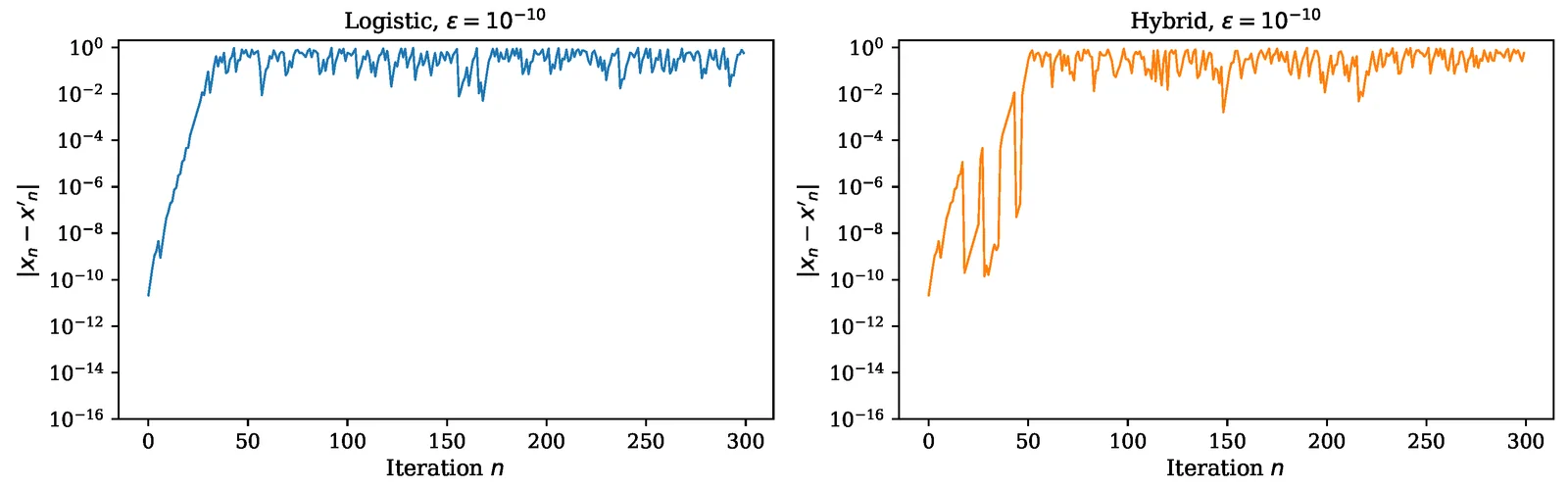
Trajectory divergence for a $10^{-10}$ initial separation.

**Figure 9:**
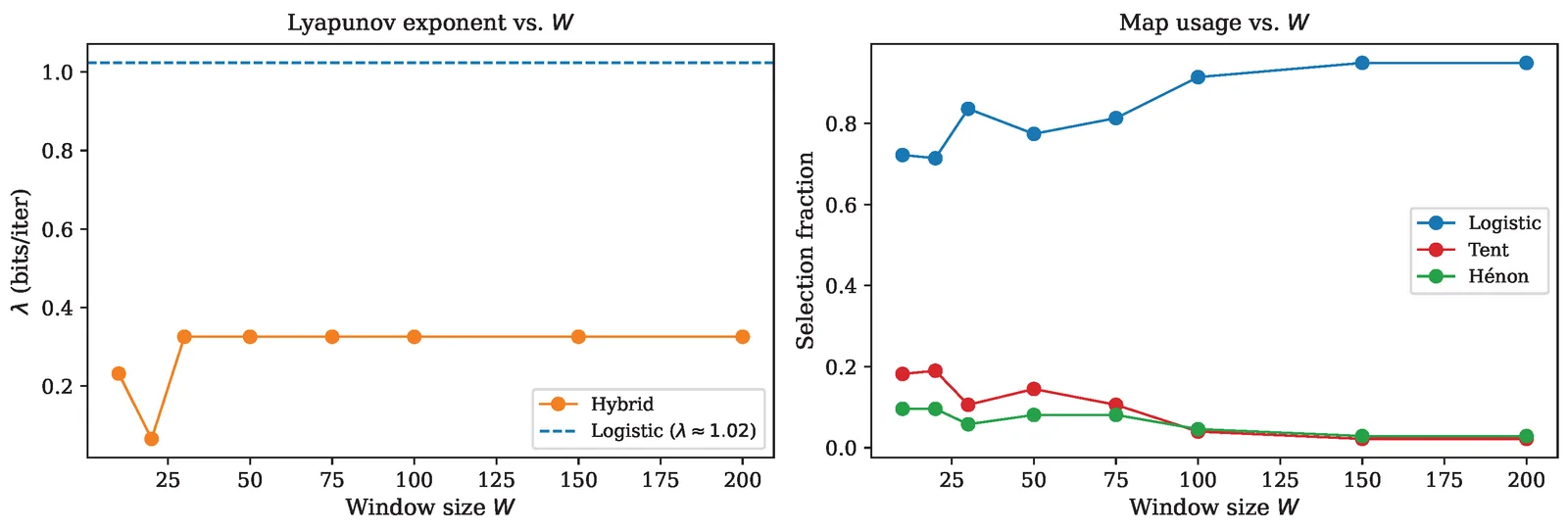
Lyapunov exponent (**left**) and selection fractions (**right**) vs. *W*.

**Figure 10:**
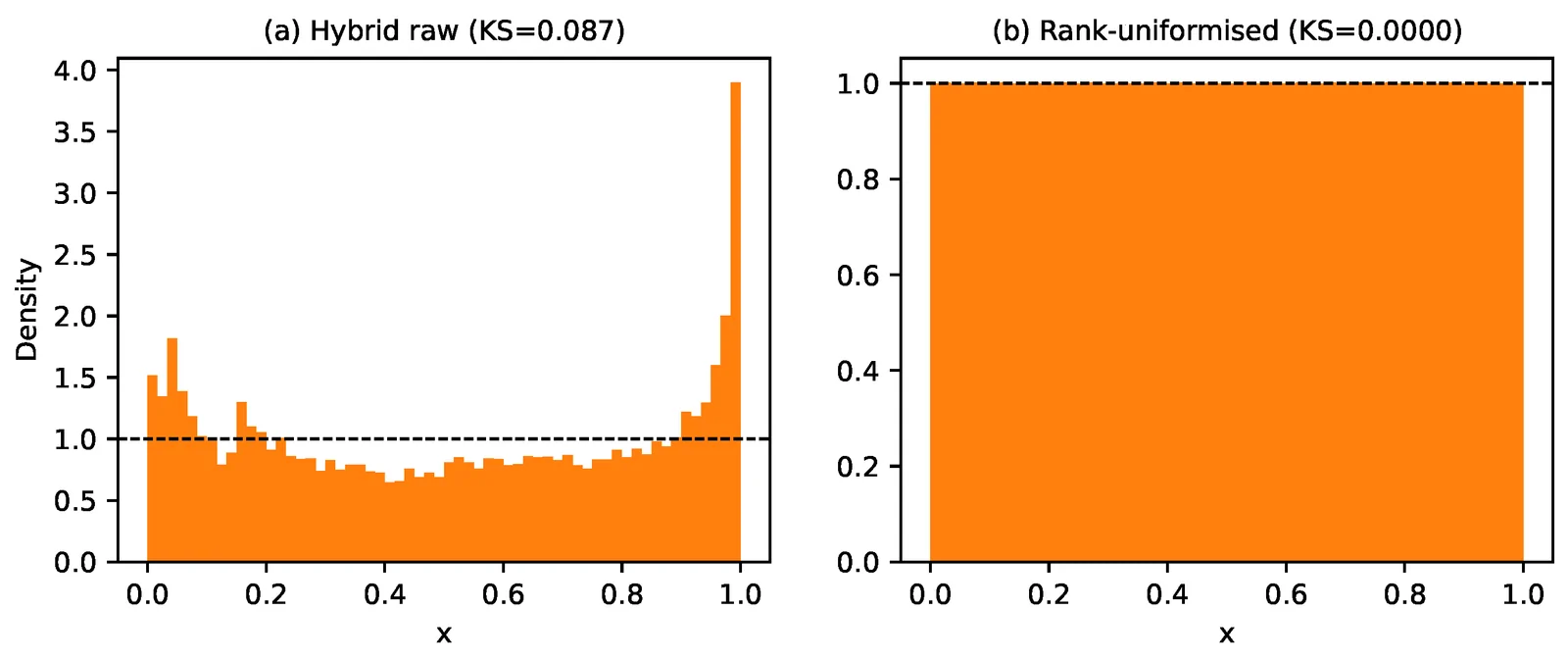
Hybrid marginal before (**a**) and after (**b**) rank-uniformisation.

**Table 1:** Wall-clock times (single-threaded Python 3.11, Intel i7-12700H).

Experiment	*N*	Time (s)
Default run (5 generators + statistics)	20,000	7.8
NIST subset (320,000 bits)	20,000	1.2
Lyapunov sweep (10×W, 50 ICs)	1000/run	42
Full pipeline (all figures + JSON)	—	51

**Table 2:** Statistical comparison (N=20,000; hybrid W=50; entropy at W=100).

Generator	KS Stat	Mean ${\hat{H}}_{n}$ (bits)	Std ${\hat{H}}_{n}$	Max $|\rho_{k}|$, k=1–10
Logistic	0.1193	3.745	0.082	0.1335
Tent	0.0278	3.879	0.064	0.0626
Hénon	0.1131	3.844	0.056	0.1680
Hybrid	0.0873	3.808	0.085	0.1248
Mersenne Twister	0.0054	3.885	0.042	0.0117

**Table 3:** NIST SP 800-22 subset (320,000 bits, α=0.01).

Stream	Monobit	Block Freq. (M=128)	Runs
Hybrid, raw	$1.7\times 10^{-21}$	1.000	0.000
Hybrid, unif.	0.611	1.000	0.685
Mersenne Tw.	0.821	0.840	0.117

## Data Availability

The raw data supporting the conclusions of this article will be made available by the authors on request.
